# Genomes in a bottle: creating standard reference materials for genomic variation - why, what and how?

**DOI:** 10.1186/gb-2011-12-s1-p31

**Published:** 2011-09-19

**Authors:** Justin M Zook, Marc Salit

**Affiliations:** 1 Biochemical Science Division, National Institute of Standards and Technology, Gaithersburg, MD 20899, USA

## 

Broad clinical application of ultra-high-throughput sequencing is imminent. In a few notable cases, actionable information has been discovered from sequencing, and the number of such cases is likely to increase. At present, there are no widely accepted genomic standards or quantitative performance metrics. These are needed to achieve the confidence in measurement results that is expected for sound, reproducible research and regulated applications. The National Institute of Standards and Technology (NIST) has been approached about considering development in this area by several commercial entities and regulatory agencies. There is great enthusiasm for translation of sequencing from the research community to clinical practice, and standards that can be used to inform confidence in measurement results (for instance, through validation studies, proficiency testing and routine quality assurance) may be an enabling factor in that goal.

NIST is currently gathering input from the genomics community about which reference materials and data would be useful. For example, NIST and the Coriell Institute for Medical Research may develop genomic reference material from cell lines from families that have already been characterized by a variety of sequencing methods (for example, the cell line from which NA12878 DNA is derived). In addition, we may build synthetic DNA constructs to test specific questions about measuring different types of variants or combinations of variants in different genomic contexts. For example, we might create pairs of constructs with single nucleotide polymorphisms, indels and/or structural variants in GC- or AT-rich regions or repeat regions. To ensure the design of appropriate standards, we are interested in discussing the design and application of genomic reference materials with any interested parties.

